# Multicomponent Intervention for Patients Admitted to an Emergency Unit for Suicide Attempt: An Exploratory Study

**DOI:** 10.3389/fpsyt.2017.00188

**Published:** 2017-09-27

**Authors:** Sebastien Brovelli, Yves Dorogi, Adam-Scott Feiner, Philippe Golay, Friedrich Stiefel, Charles Bonsack, Laurent Michaud

**Affiliations:** ^1^Service of Liaison Psychiatry, Department of Psychiatry, Lausanne University Hospital (CHUV), Lausanne, Switzerland; ^2^Department of Emergency Medicine, Lausanne University Hospital, Lausanne, Switzerland; ^3^Service of Community Psychiatry, Department of Psychiatry, Lausanne University Hospital, Lausanne, Switzerland

**Keywords:** suicide, suicide attempt, emergency unit, phone contacts, intervention, pilot study

## Abstract

Suicide is a major cause of premature deaths worldwide and belongs to the top priority public health issues. While suicide attempt is the most important risk factor for completed suicide, intervention for suicide attempters (SA) have produced mixed results. Since an important proportion of SA request medical care, emergency units (EU) are an opportune setting to implement such interventions. This exploratory study evaluated the feasibility and acceptability of a multicomponent intervention for SA admitted to an EU. The intervention consisted of coordination by a case manager of a joint crisis plan (JCP), an early meeting with relatives and the existing care network, as well as phone contacts during 3 months after suicide attempt. Among 107 SA admitted to the emergency unit during the study period, 51 could not be included for logistical reason, 22 were excluded, and intervention was offered to 34. Of these, 15 refused the intervention, which was thus piloted with 19 SA. First-time attempters most frequently declined the intervention. Feasibility and acceptability of phone contacts and case manager were good, while JCPs and meetings were difficult to implement and perceived as less acceptable. Refusal pattern questions the global acceptability and is discussed: JCPs and meetings will have to be modified in order to improve their feasibility and acceptability, especially among first-time attempters.

## Introduction

Suicide is a major public health issue: 804,000 suicide deaths were estimated worldwide in 2012, suicide being the 15th leading cause of death (1) and self-harm accounting for 36,654 disability-adjusted life years, thus being in the 20th leading causes of disability and premature death (2). Suicide attempts are approximately 20 times more frequent than completed suicide (1) and having attempted suicide is the strongest predictor of suicide (3–6). Between 0.5 and 2% of suicide attempters (SA) died by suicide during the year following their suicide attempt (7), more than 5% die of suicide between 9 and 18 years after (7–9) and around 10% during long-term follow-up (5); suicide risk is thus hundred times higher in SA than in the general population (7, 10). In Switzerland, it is estimated that only 10,000 of the yearly 15,000–25,000 suicide attempts seek medical treatment (11).

While an unknown proportion of suicide attempts remain undetected, many SA require medical care and are admitted in an emergency unit (EU) (12, 13). Although the majority of suicidal patients seen in EU are referred for outpatient follow-up after discharge, only 25–50% actually attend outpatient appointments within 1 month after the attempt (14). EUs and psychiatric consultation-liaison services play a key role in identifying these patients and enhancing their adherence to further treatment. Interventions have to be timely, fast, and efficient because length of stay in EU is often very short.

A large range of post suicide attempt interventions have been conceived. Minimal interventions based on repeated follow-up contacts by phone, postal letters, postcards, e-mails, or texting appear to reduce suicidal behavior and can be targeted to specific patient populations (15–20). Furthermore, post-discharge follow-up calls after a suicide attempt were found to be relatively inexpensive and effective (21). More costly psychosocial interventions do not seem to prevent completed suicide (22). On the other hand, psychotherapy showed promising results in reducing repeated suicidal behavior (23, 24) especially in high-risk SA (25). A systematic review showed that in young patients (15–24 years), transition interventions (EU to post-EU care) seemed to reduce suicide-related outcomes and improve post-EU treatment adherence, compared to interventions initiated only after EU discharge and to EU-based interventions (26). In adults, active contact and follow-up seem to reduce the risk of a further SA at 12 months (16, 27).

We did not identify any intervention based on this background and also including the clinical meaningful experience of closely involving relatives and existing care networks in the management. Therefore, we aimed to develop and evaluate a multicomponent intervention with regard to its feasibility, acceptability, and clinical outcomes. The intervention seeks to improve the usual follow-up of SA admitted in EU. The second objective was to compare the profile of patients that were included and excluded of the study and also to compare the characteristics of patients who accepted or refused the intervention.

## Materials and Methods

### Study Design

Exploratory study of the feasibility, acceptability, and effects of a multicomponent intervention for SA.

### Patient Population

Participants were SA (18–65 years) admitted to the emergency unit of Lausanne University Hospital, Switzerland, for whom a psychiatric consultation was requested from 8:00 a.m. to 5:00 p.m. between Monday and Friday. As no specific resource was allocated for this pilot study, its implementation relied on our usual psychiatric staff. SA evaluated during the evenings, nights, and weekend could, therefore, not be considered for inclusion and only one SA per day could be included in the study. Prisoners and non-French speaking patients were excluded. Suicide attempt was defined as any non-fatal intentional act of self-poisoning or self-injury, irrespective of degree of suicidal intent or other types of motivation (28), leading to EU admission. The SA who agreed to participate in the study were invited to sign an informed consent form.

### Intervention

**Figure 1:**
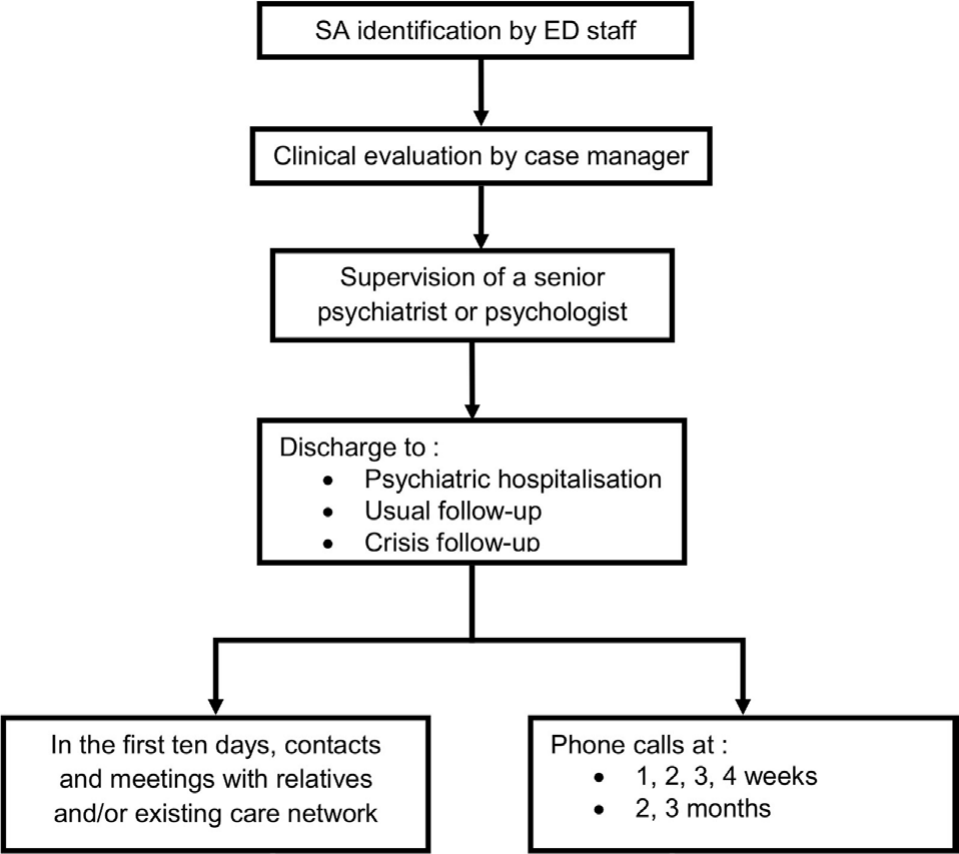
Intervention.

The intervention specifically addressed SA in the EU and was coordinated by a case manager specialized in mental health care and working in our Emergency Psychiatric Unit. It consisted of several components issued from a literature review of best practices (Figure 1): coordination by a case manager (CCM) (19, 29); joint crisis plan (JCP) (30–32); early meeting with relatives and existing care network (EM) (33, 34); and phone contacts during 3 months after suicide attempt (PC) (15–19, 27, 35).

Three different CCM participated in the intervention, all of them having more than 5 years experience in the psychiatric emergency setting. They aimed to offer the SA a continuous relationship with a single person during the whole intervention. Case managers first performed a clinical evaluation of the SA under supervision of a senior staff member (psychiatrist or psychologist), to whom a comprehensive assessment of the suicidal crisis, including the current and recent (8 weeks) suicidal events as well as the immediate and future suicidal thoughts (36), were presented. The context and meaning of the current crisis were also explored and a JCP was developed, together with the SA. The JCP aimed to increase (i) the patient’s implication in the identification of the stressful elements leading to suicide attempt and (ii) the exploration of potential coping strategies for a subsequent crisis and (iii) to make further therapeutic decisions easier.

After clinical evaluation, participants were discharged from the EU to inpatient psychiatric care, referred to their previous therapist or referred to a crisis follow-up clinic, which is part of our Emergency Psychiatric Unit. The case manager also contacted relatives and suggested a meeting with him and a senior clinician if the SA agreed and no major counter indications were present, with the aim to share thoughts and emotions evoked by the suicidal event and provide education on suicidal crisis issues. EM was also contacted in order to discuss the suicidal event and to reach a consensus relative to further appropriate clinical management and eventually arrange a meeting.

The final part of the intervention consisted of weekly phone contacts for 1 month, and then monthly for another 2 months. Case manager called SA and get back over the period since the last contact with him/her. They offered listening and attention to the SA and answered any relevant questions. They attempted to enhance adherence to treatment and, if required, they motivated and directed the SA to seek further assistance or care (19).

### Measures

Baseline data on sociodemographic (age, gender, origin, civil status, children, education, occupation, finances, legal representation, household composition) and clinical data (person who referred, past history of treatment, past history of suicide attempt, type of suicide attempt, post suicide attempt follow-up) were assessed for 107 SA. ICD-10 diagnosis was assessed by a psychiatric fellow under the supervision of a senior clinician. In addition, hopelessness was assessed at baseline by means of the French version of the Beck Hopelessness Scale (BHS) (37), a 20-item self-report instrument designed to measure negative expectations about the future. Total scores range from 0 to 20, with a score higher than eight indicating levels of hopelessness associated with an increased risk of suicide.

Feasibility was assessed by means of regular meetings with the project team during which difficulties of the intervention were discussed.

Acceptability was measured by means of satisfaction rated at baseline and at the end of the intervention. We used the Client Satisfaction Questionnaire (CSQ-8) (38), a 8-item self-report instrument designed to evaluate the service/treatment provided to clients/patients. Each item is phrased as a four-point anchored answer without a neutral position. We added two open-ended questions to this questionnaire: “*What did you most appreciate in your contacts with the psychiatric team*?” and “*What do you think could be improved with regard to these contacts?*” This questionnaire was administered at T0 by CCM and at the end of intervention by phone by a member of the research team.

During the phone follow-up, the CCM collected other clinical variables such as drop outs, suicide reattempts, and adherence to treatment.

Table 1 summarizes the different components of the intervention and the different instruments.

**Table 1:** Components and instruments of the intervention.

Time	Intervention	Measures
T0	– Information and informed consent – Exploration of the suicidal crisis – Elaboration of a treatment plan – Anticipation and preparation of possible future suicidal crises	– Suicide attempters target, collection of sociodemographic and clinical data – Beck Hopelessness Scale (37) – Joint Crisis Plan (31, 32) – Immediate Client Satisfaction Questionnaire (CSQ-8) (38)
T1	Contact and/or meeting with relatives and/or the care network	
T2–T7	Phone contacts by the CCM	– Collection of clinical data: suicidal reattempts rate, treatment adherence
T8	Collections by a member of the research team (phone contact)	– CSQ-8 at 3 months

### Statistical Analysis

Comparisons between groups were performed with independent *t*-tests for continuous variables and Mann–Whitney *U*-tests for ordinal variables. For nominal variables, analyses were performed with Pearson’s Chi-Square tests or Fisher Exact tests when appropriate. All statistical tests were two-tailed and significance was determined at the 0.05 level. All statistical analyses were performed with IBM SPSS version 22.

## Results

**Figure 2:**
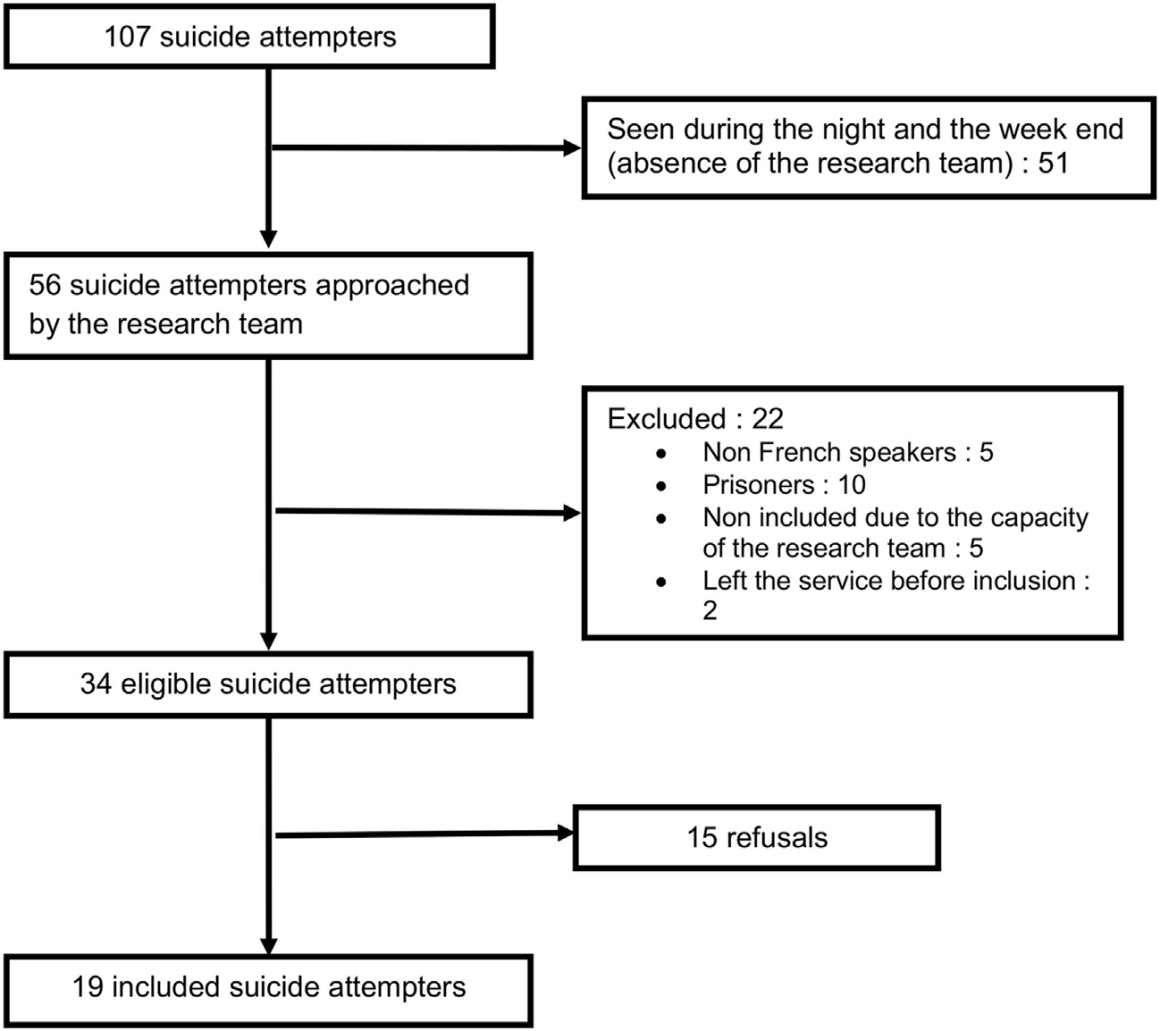
Participants.

Between January 14th and June 30th, 2014, a psychiatric consultation was requested for 107 SA. For logistical reasons, 51 SA were not included, as mentioned above, and 22 were excluded. The intervention was proposed to 34 SA and accepted by 19. Reasons for refusal were refusal of an additional psychiatric intervention and unwillingness to participate in a study (Figure 2).

### Clinical and Demographic Baseline Characteristics

Clinical and demographic characteristics of included patients (*N* = 19) were compared to those who were not approached (*N* = 51), excluded (*N* = 22), and those who refused (*N* = 15) (all referred to as non-included) (see Table 2). An important proportion (47%) of SA was not included because of the intervention scheduling (only available during working days). Included patients significantly differed from non-included patients on several dimensions: they were more often female [84 vs 50%, χ^2^(1) = 7.4, *p* = 0.006], more often referred by a relative (68 vs 40%, *p* = 0.041), they more often had financial problems [78 vs 52%, χ^2^(1) = 3.8, *p* = 0.049], and were more often under curatorship (26 vs 3%, *p* = 0.005).

**Table 2:** Comparison between patients that were included and excluded of the study.

Variable		Total patients *N* = 107	Included in study *N* = 19	Excluded of study *N* = 88	Statistic	*p*-Value
Age (years)	M (SD)	36.5 (11.3)	34.4 (9.6)	36.9 (11.7)	*t*(105) = −0.893	0.374
Gender (% male)	% (*n*)	43.9 (47)	15.8 (3)	50.0 (44)	χ^2^(1) = 7.425	0.006
Diagnostic						
Alcohol F1	% (*n*)	15.5 (16)	15.8 (3)	15.5 (13)	a	0.066
Drugs F11-F19	% (*n*)	11.7 (12)	5.3 (1)	13.1 (11)		
Schizophrenia F2	% (*n*)	5.8 (6)	10.5 (2)	4.8 (4)		
Mania F3-M	% (*n*)	1.9 (2)	0.0 (0)	2.4 (2)		
Depression F3-D	% (*n*)	27.2 (28)	5.3 (1)	32.1 (1)		
Neurotic, stress-related disorder F4	% (*n*)	23.3 (24)	36.8 (7)	20.2 (17)		
Personality disorder F6	% (*n*)	14.6 (15)	26.3 (5)	11.9 (10)		
Sent by						
Patient	% (*n*)	10.3 (11)	10.5 (2)	10.2 (9)	a	0.041
Relative	% (*n*)	44.9 (48)	68.4 (13)	39.8 (35)		
Health professional	% (*n*)	27.1 (29)	21.1 (4)	28.4 (25)		
Other	% (*n*)	17.8 (19)	0.0 (0)	21.6 (19)		
Origin (% swiss)	% (*n*)	43.9 (47)	57.9 (11)	40.9 (36)	χ^2^(1) = 1.830	0.176
Living						
Alone	% (*n*)	22.4 (24)	26.3 (5)	21.6 (19)	a	0.223
With others (private)	% (*n*)	54.2 (58)	57.9 (11)	53.4 (47)		
Institution	% (*n*)	10.3 (11)	15.8 (3)	9.1 (8)		
Other	% (*n*)	13.1 (14)	0.0 (0)	15.9 (14)		
Marital status						
Single	% (*n*)	51.4 (55)	63.2 (12)	48.9 (43)	a	0.407
Married	% (*n*)	23.4 (25)	10.5 (2)	26.1 (23)		
Registered partnership	% (*n*)	1.9 (2)	0.0 (0)	2.3 (2)		
Divorced	% (*n*)	16.8 (18)	26.3 (5)	14.8 (13)		
Separated	% (*n*)	5.6 (6)	0.0 (0)	6.8 (6)		
Widowed	% (*n*)	0.9 (1)	0.0 (0)	1.1 (1)		
With children (% yes)	% (*n*)	50.5 (50)	36.8 (7)	53.8 (43)	χ^2^(1) = 1.756	0.185
Education						
In progress	% (*n*)	7.9 (5)	5.3 (1)	7.9 (5)	a	0.803
Interrupted	% (*n*)	4.8 (3)	5.3 (1)	4.8 (3)		
Compulsory education	% (*n*)	25.4 (16)	31.6 (6)	25.4 (16)		
Apprenticeship	% (*n*)	27.0 (17)	31.6 (6)	27.0 (17)		
High school	% (*n*)	3.2 (2)	5.3 (1)	3.2 (2)		
Professional/commercial school	% (*n*)	14.3 (9)	15.8 (3)	14.3 (9)		
University	% (*n*)	15.9 (10)	5.3 (1)	15.9 (10)		
Other	% (*n*)	1.6 (1)	0.0 (0)	1.6 (1)		
Occupation						
In training	% (*n*)	6.5 (7)	15.8 (3)	4.5 (4)	a	0.052
Working full time	% (*n*)	15.9 (17)	10.5 (2)	17.0 (15)		
Working part time	% (*n*)	12.1 (13)	26.3 (5)	9.1 (8)		
Occupational	% (*n*)	4.7 (5)	0.0 (0)	5.7 (5)		
Unemployed	% (*n*)	42.1 (45)	21.1 (4)	46.6 (41)		
Retired	% (*n*)	0.9 (1)	0.0 (0)	1.1 (1)		
Disability annuitant	% (*n*)	17.8 (19)	26.3 (5)	15.9 (14)		
Finances (% problematic)	% (*n*)	57.7 (45)	77.8 (14)	51.7 (31)	χ^2^(1) = 3.868	0.049
Legal representation						
None	% (*n*)	90.2 (83)	73.7 (14)	94.5 (69)	a	0.005
Citizen curatorship	% (*n*)	7.6 (7)	26.3 (5)	2.7 (2)		
Professional curatorship	% (*n*)	1.1 (1)	0.0 (0)	1.4 (1)		
Parents	% (*n*)	1.1 (1)	0.0 (0)	1.4 (1)		
Past history of treatment						
None	% (*n*)	20.6 (22)	10.5 (2)	22.7 (20)	a	0.116
General practitioner	% (*n*)	13.1 (14)	21.1 (4)	11.4 (10)		
Psychiatrist/psychologist	% (*n*)	65.4 (70)	63.2 (12)	65.9 (58)		
Other	% (*n*)	0.9 (1)	5.3 (1)	0.0 (0)		
Past history of suicide attempt						
None	% (*n*)	40.2 (41)	26.3 (5)	43.4 (36)	*U* = 631.500	0.157
Once	% (*n*)	21.6 (22)	26.3 (5)	20.5 (17)		
Twice	% (*n*)	7.8 (8)	5.3 (1)	8.4 (7)		
Thrice	% (*n*)	4.9 (5)	5.3 (1)	4.8 (4)		
More than three	% (*n*)	25.5 (26)	36.8 (7)	22.9 (19)		
Suicide attempt methodology						
Drug poisoning	% (*n*)	64.2 (68)	100.0 (19)	56.3 (49)	a	0.074
Other substances poisoning	% (*n*)	4.7 (5)	0.0 (0)	5.7 (5)		
Sharp object	% (*n*)	17.0 (18)	0.0 (0)	20.7 (18)		
Firearm	% (*n*)	0.9 (1)	0.0 (0)	1.1 (1)		
Jumping	% (*n*)	0.9 (1)	0.0 (0)	1.1 (1)		
Hanging	% (*n*)	2.8 (3)	0.0 (0)	3.4 (3)		
Several methods	% (*n*)	6.6 (7)	0.0 (0)	8.0 (7)		
Other	% (*n*)	2.8 (3)	0.0 (0)	3.4 (3)		
Post suicide attempt follow-up						
None	% (*n*)	2.8 (3)	0.0 (0)	3.4 (3)	a	0.184
Liaison psychiatry	% (*n*)	15.9 (17)	31.6 (6)	12.5 (11)		
Treating general practitioner	% (*n*)	0.9 (1)	0.0 (0)	1.1 (1)		
Treating psychiatrist/psychologist	% (*n*)	32.7 (35)	47.4 (9)	29.5 (26)		
Other treating health professional	% (*n*)	5.6 (6)	0.0 (0)	6.8 (6)		
Liaison psychiatry with university hospital	% (*n*)	3.7 (4)	0.0 (0)	4.5 (4)		
Voluntary psychiatric admission	% (*n*)	23.4 (25)	21.1 (4)	23.9 (21)		
Involuntary psychiatric admission	% (*n*)	14.0 (15)	0.0 (0)	17.0 (15)		
Non-psychiatric admission	% (*n*)	0.9 (1)	0.0 (0)	1.1 (1)		

Table 3 shows the comparison between included patients (*N* = 19) and those who refused (*N* = 15): more first-time attempters refused the intervention (60 vs 26%; *p* = 0.044). Only SA with self poisoning accepted the intervention, hence 27% of those who refused used several other methods (drug and other substances poisoning, cutting and jumping from a height; *p* = 0.029). People who had children also more frequently refused the intervention (79 vs 37%, *p* = 0.017). All other sociodemographic characteristics were similar between groups. The average score of the BHS, completed by the 19 included SA, reached 8.2 (SD 4.2) on average.

**Table 3:** Comparison between patients that accepted or refused the intervention.

Variable		Total patients *N* = 34	Accepted *N* = 19	Refused *N* = 15	Statistic	*p*-Value
Age (years)	M (SD)	36.9 (10.4)	34.4 (9.6)	40.1 (10.7)	*t*(32) = −1.653	0.108
Gender (% male)	% (*n*)	23.5 (8)	15.8 (3)	33.3 (5)	a	0.417
Diagnostic						
Alcohol F1	% (*n*)	14.7 (5)	15.8 (3)	13.3 (2)	a	0.659
Drugs F11–F19	% (*n*)	8.8 (3)	5.3 (1)	13.3 (2)		
Schizophrenia F2	% (*n*)	8.8 (3)	10.5 (2)	6.7 (1)		
Mania F3-M	% (*n*)	2.9 (1)	0.0 (0)	6.7 (1)		
Depression F3-D	% (*n*)	11.8 (4)	5.3 (1)	20.0 (3)		
Neurotic, stress-related disorder F4	% (*n*)	29.4 (10)	36.8 (7)	20.0 (3)		
Personality disorder F6	% (*n*)	23.8 (8)	36.8 (7)	20.0 (3)		
Sent by						
Patient	% (*n*)	11.8 (4)	10.5 (2)	13.3 (2)		0.689
Relative	% (*n*)	61.8 (21)	68.4 (13)	53.3 (8)		
Health professional	% (*n*)	26.5 (9)	21.1 (4)	33.3 (5)		
Origin (% Swiss)	% (*n*)	50.0 (17)	57.9 (11)	40.0 (6)	χ^2^(1) = 1.074	0.300
Living						
Alone	% (*n*)	32.4 (11)	26.3 (5)	40.0 (6)	a	0.300
With others (private)	% (*n*)	58.8 (20)	57.9 (11)	60.0 (9)		
Institution	% (*n*)	8.8 (3)	15.8 (3)	0.0 (0)		
Marital status						
Single	% (*n*)	52.9 (18)	63.2 (12)	40.0 (6)	a	0.325
Married	% (*n*)	11.8 (4)	10.5 (2)	13.3 (2)		
Divorced	% (*n*)	29.4 (10)	26.3 (5)	33.3 (5)		
Separated	% (*n*)	5.9 (2)	0.0 (0)	13.3 (2)		
With children (% yes)	% (*n*)	54.5 (18)	36.8 (7)	78.6 (11)	χ^2^(1) = 5.661	0.017
Education						
In progress	% (*n*)	3.2 (1)	5.3 (1)	0.0 (0)	a	0.648
Interrupted	% (*n*)	6.5 (2)	5.3 (1)	8.3 (1)		
Compulsory education	% (*n*)	25.8 (8)	31.6 (6)	16.7 (2)		
Apprenticeship	% (*n*)	35.5 (11)	31.6 (6)	41.7 (5)		
High school	% (*n*)	3.2 (1)	5.3 (1)	0.0 (0)		
Professional/commercial school	% (*n*)	12.9 (4)	15.8 (3)	8.3 (1)		
University	% (*n*)	12.9 (4)	5.3 (1)	25.0 (3)		
Occupation						
In training	% (*n*)	8.8 (3)	15.8 (3)	0.0 (0)	a	0.663
Working full time	% (*n*)	14.7 (5)	10.5 (2)	20.0 (3)		
Working part time	% (*n*)	26.5 (9)	26.3 (5)	26.7 (4)		
Unemployed	% (*n*)	20.6 (7)	21.1 (4)	20.0 (3)		
Disability annuitant	% (*n*)	29.4 (10)	26.3 (5)	33.3 (5)		
Finances (% problematic)	% (*n*)	70.0 (21)	77.8 (14)	58.3 (7)	a	0.418
Legal representation						
None	% (*n*)	82.4 (28)	73.7 (14)	93.3 (14)	a	0.053
Citizen curatorship	% (*n*)	14.7 (5)	26.3 (5)	0.0 (0)		
Professional curatorship	% (*n*)	2.9 (1)	0.0 (0)	6.7 (1)		
Past history of treatment						
None	% (*n*)	14.7 (5)	10.5 (2)	20.0 (3)	a	0.766
General practitioner	% (*n*)	17.6 (6)	21.1 (4)	13.3 (2)		
Psychiatrist/psychologist	% (*n*)	64.7 (22)	63.2 (12)	66.7 (10)		
Other	% (*n*)	2.9 (1)	5.3 (1)	0.0 (0)		
Past history of suicide attempt						
None	% (*n*)	41.2 (14)	26.3 (5)	60.0 (9)	*U* = 87.500	0.044
Once	% (*n*)	23.5 (8)	26.3 (5)	20.0 (3)		
Twice	% (*n*)	2.9 (1)	5.3 (1)	0.0 (0)		
Thrice	% (*n*)	5.9 (2)	5.3 (1)	6.7 (1)		
More than three	% (*n*)	26.5 (9)	36.8 (7)	13.3 (2)		
Suicide attempt methodology						
Drug poisoning	% (*n*)	88.2 (30)	100.0 (19)	73.3 (11)	a	0.029
Other substances poisoning	% (*n*)	2.9 (1)	0.0 (0)	6.7 (1)		
Sharp object	% (*n*)	5.9 (2)	0.0 (0)	13.3 (2)		
Several methods	% (*n*)	2.9 (1)	0.0 (0)	6.7 (1)		
Post suicide attempt follow-up						
Liaison psychiatry	% (*n*)	26.5 (9)	31.6 (6)	20.0 (3)	a	0.294
Treating psychiatrist/psychologist	% (*n*)	44.1 (15)	47.4 (9)	40.0 (6)		
Voluntary psychiatric admission	% (*n*)	20.6 (7)	21.1 (4)	20.0 (3)		
Involuntary psychiatric admission	% (*n*)	8.8 (3)	0.0 (0)	20.0 (3)		

### Feasibility of Intervention

CCM was perceived most of the time as satisfying by the research team. However, JCP was observed to be difficult to complete during EU stay, because of the acute psychiatric state and the numerous other tasks required during the EU hospitalization (comprehensive clinical assessment, information on study, baseline data collection). Therefore, the JCP’s documentation was rarely completed.

Six joint meetings with the SA and their relatives were organized, four after discharge and two in the EU. The relatives were motivated to come in EU, but reported difficulties in participating in a meeting a few days after the suicide attempt. Meetings with relatives were rare and could not be organized as systematically as expected. A meeting with the existing care network was organized only once.

The phone follow-up revealed to be feasible. Only 20 of the 102 planned calls could not be realized because patients did not answer despite three attempts. Case Managers reported that a trustful relationship with an adequate therapeutic distance could be built, fostering the development and continuity of follow-up.

### Acceptability of Intervention

The CSQ-8 was completed by 14 SA after the intervention in the EU. The average score of the total of points (max sum score = 32) of the questionnaire was 27.8 (SD 2.8). Open-ended questions revealed that elements, such as “empathy,” “availability,” “listening,” and “attention” were valued by patients. Answers to the question about what could be improved revealed a perceived lack of time and of intimacy during the intervention and difficulties of collaboration with EU staff.

The CSQ-8 was completed by phone for 15 SA at the end of the intervention: the average score was 25.4 (SD 4.9). The intervention was described as supportive and respectful, facilitating EU discharge and providing structure and support during the 3 months following the suicide attempt. The weekly rhythm of phone contacts was described as satisfying, but the diminished rhythm between phone contacts at 1 and 2 months was perceived as abrupt.

### Clinical Outcomes

Among the 19 SA included in the study, 14 terminated the intervention, four dropped out (one after the meeting in the EU, one after 1 month and, two after 2 months), and one (5%) died by suicide. Twelve of the 19 included patients did not reattempt suicide during the intervention, three (16%) reattempted suicide (one once, one twice and one three times). The SA who died by suicide was diagnosed with major depression. He accepted crisis follow-up, but declined inpatient psychiatric care. He missed no appointment during follow-up. After reporting a favorable evolution during a last meeting, he hung himself. BHS score was three for the patient who died by suicide, and 11 for the three reattempters.

## Discussion

Results of this exploratory study indicate that a 5/7 day intervention can address less than half of all SA admitted to an EU. When the intervention was proposed to SA, it was less frequently accepted by first-time SA (FTA), SA with other methods than drug self poisoning and people with children. An acceptance bias might explain the low rate of FTA: patients who already had treatment before the suicide attempt might more easily accept a new type of intervention. Furthermore, it was easier to include SA with a pre-existing relationship with the service, than establishing a therapeutic alliance with FTA. An identification bias might also have favored the systematic reporting of SA with a psychiatric diagnosis, since FTA without a psychiatric diagnosis might have been trivialized (39). Regarding the suicide method, some types, such as cutting, might have been considered less serious and prematurely discharged from EU (39, 40). In addition, drug poisoning involves a longer stay in EU, which could have favored inclusion in the study. For patients using methods identified in the non-included group (such as hanging, gassing, jumping from a height, or using a firearm) who have a worse prognosis in term of subsequent completed suicide (41), specific strategies to favor inclusion should be developed. Interestingly, we found that SA with children were more likely to decline our intervention. We did not identify any previous research regarding this specific aspect; persons with children might have more difficulties to engage in care, for organizational reasons or because of a desire to protect their children from becoming aware of their difficulties.

These results concerning included patients provide interesting and unexpected information with regard to feasibility. They raise the issue of a better characterization of SA, which could contribute to define specific, targeted interventions for these subgroups. Mendez-Bustos and colleagues (42) systematic review sought to identify key demographic, psychological, and clinical variables associated with suicide reattempters. They found that suicide reattempters, compared to single attempters showed higher rates of unemployment, unmarried status, diagnoses of mental disorders, suicidal ideation, stressful life events, and family history of suicidal behavior. Other studies found that major repeaters or multiple SA constitute a specific subgroup of severely disturbed patients who require particular attention (43–49). We also know that survivors of a first suicide attempt are at increased odds of having psychiatric morbidity and/or comorbidity (50) and that the age at onset of first suicide attempt characterizes different subpopulations (51, 52). Finally, hypomanic symptoms by FTA predict multiple future suicide attempts (53).

Concerning the feasibility of the intervention, systematic meetings with relatives or care network were difficult to organize. They represented a challenge during the emergency situation and a significant change from usual practice. Considering these difficulties, further interventions should adapt their use and they should probably be reserved to specific situations such as young SA (33). Additionally, JCP was founded difficult to complete in the EU, particularly due to time constraints and the patients suboptimal physical and psychiatric condition. SA appreciated phone contacts, which were the least expensive component of the intervention, in terms of time and cost (21).

Regarding acceptability of the intervention, satisfaction was high for the SA who had access to the intervention. The contact with the case manager was appreciated beyond the intervention in EU and there were few drop outs during follow-up, SA emphasizing the benefits of the continuity of the intervention. Both SA and case managers felt that that the short stay in EU limited the initial meeting’s ability to build therapeutic alliance and engage patients for follow-up.

A minimum initial EU sojourn of 24 h would facilitate initial contact and intervention quality. Alternately, systematic meeting in SA’s home after a rapid EU discharge might be more feasible and efficient. Such a meeting could be based on previous experiences with home interventions (14, 54, 55) and could also be used to complete the JCP, thus addressing the above mentioned difficulty for completing JCP in EU.

The small size of our sample and the absence of control group do not allow to assess the efficacy of the intervention. From a descriptive point of view, the outcomes seem to be in line with other interventions: our sample showed rates of fatal (5%) and non-fatal (16%) suicide attempts similar to those reported in other studies (5, 7–10, 56, 57). One included SA died by suicide, reflecting the high suicide risk in this population and the difficulty of preventing completed suicide, even with intensive crisis intervention. As found by others (58, 59), the BHS had a weak predictive value for suicidal recurrence and completed suicide and should, therefore, be used with caution in these situations.

### Limitations

This pilot study was limited by the small sample size and the large proportion of SA who presented out of the inclusion times (nights and weekends). Furthermore, some components of intervention, in particular, family meetings proved to be much more difficult to organize than initially expected. 24/7 availability of our intervention would greatly enhance patient recruitment and engagement of patients in our proposed treatment.

Another limitation is the definition of the term of “suicide attempt.” Silverman et al. (60) showed that the variability in terminology and definitions used in the suicide literature strongly affects the collected data and hampers comparison, extrapolation, and generalization. The term “suicide attempt” can encompass a wide range of non-fatal self-inflicted behaviors. In this study, we considered all intentional self-harm as suicide attempt, following the approach favored in the UK (28). Suicidal intent is regarded as a dimensional rather than a categorical concept within the definition of “self-harm” (28). The choice of this definition of suicide attempt may have had a considerable influence on the recruited patients.

## Conclusion

Suicide attempters remain a population at high risk of completed suicide who warrant intensive and systematic follow-up. In this exploratory study, JCP and EM showed lower feasibility and acceptability than CCM and PC and should be modified in order to improve intervention. Our final goal is to conceptualize a better intervention and test it using a Randomized Controlled Trial. As a first step, we are presently conducting a qualitative addendum of the present study and aim to use SA’s point of view to improve our intervention. In addition, FTA and users of other methods than drug poisoning were more difficult to engage in an intervention. We were authorized by the Ethics Committee to include in our addendum SA who refused our intervention and these engagement issues will also be explored with them. Other developments and research should also specifically address these populations.

## Ethics Statement

This study was carried out in accordance with the recommendations of the cantonal ethic committee on human research of Vaud with written informed consent from all subjects. All subjects gave written informed consent in accordance with the Declaration of Helsinki. The protocol was approved by the ethic committee on human research of Vaud. Name of Ethics Committee: Commission cantonale (VD) d’éthique de la recherche sur l’être humain, avenue de Chailly 23, 1012 Lauanne. Approval number: Protocole 411/2013.

## Author Contributions

Substantial contributions to conception and design (SB, YD, FS, CB, A-SF, and LM), acquisition of data (SB, YD, and LM), analysis (SB, PG, and LM), and interpretation (SB, PG, YD, and LM) of data; drafting of the article (SB, PG, and LM) and revising it critically for important intellectual content (all); final approval of the version to be submitted and any revised version (all).

## Conflict of Interest Statement

The authors declare that the research was conducted in the absence of any commercial or financial relationships that could be construed as a potential conflict of interest.
